# Preparedness for Mass Gatherings: Factors to Consider According to the Rescue Authorities

**DOI:** 10.3390/ijerph17041361

**Published:** 2020-02-20

**Authors:** Anssi Koski, Anne Kouvonen, Hilla Sumanen

**Affiliations:** 1 South-Eastern Finland University of Applied Sciences, 48220 Kotka, Finland; hilla.sumanen@helsinki.fi; 2 Faculty of Medicine, University of Helsinki, 00014 Helsinki, Finland; 3 Faculty of Social Sciences, University of Helsinki, 00014 Helsinki, Finland; anne.kouvonen@helsinki.fi; 4 Research Institute of Psychology, SWPS University of Social Sciences and Humanities, 53-238 Wrocław, Poland; 5 Centre for Public Health, Queen’s University Belfast, Belfast BT12 6BA, UK

**Keywords:** preparedness, mass gatherings, emergency medical services, rescue service, fire service, large crowd events, event organizers

## Abstract

*Background*: Mass gatherings cause a need for multi-authority preparedness in order to ensure the safety of the event participants and to minimize delays in response for emergencies. Rescue authorities are key players in the pre-planning phase; however, their own point of view regarding all aspects of preparedness for mass gatherings is not well known. The aim of this study was to investigate what factors, according to the rescue authorities, need to be considered when preparing for mass gatherings. *Method*: Semi-structured thematic interviews were carried out with the rescue authorities involved in the mass gathering planning process (*n* = 15). The transcribed material was analyzed using inductive content analysis. *Results*: Three main categories emerged from the interviews: (1) co-operation in the pre-planning phase, (2) factors to be noted in the emergency plan, and (3) actions during the event. These categories were divided into 11 generic categories, which were further divided into 42 sub-categories. *Conclusion*: Rescue authorities recognized various factors considering preparedness for mass gatherings. Knowledge considering the dispersion of operative workload during the event needs further investigation in order to facilitate the effective use of limited operative resources.

## 1. Introduction

Mass gatherings are situations or events that attract large crowds and may create potential delays in emergency response. Delays can be caused, for example, by limited access to patients or factors related to the location and environment [1]. Participants and spectators of mass gatherings also have a higher density of injuries and illnesses compared to the general population, even though they consist of generally healthier individuals than the overall population [1]. In addition, mass gatherings create a potential risk for catastrophic accidents, such as human stampedes or being a subject of violent sabotage, which may both lead to a mass casualty incident [1,2,3]; there is also a risk of infectious diseases [4,5]. In addition, mass gatherings can increase police forces’ workload [6,7,8]. Issues in fire safety of mass gatherings may cause disastrous consequences and, thus, have an impact on resources for rescue services (term used in this study, equal to fire service) [9]. These characteristics of mass gatherings create a need for careful pre-planning and preparedness, carried out by robust multi-authority co-operation together with the event organizers and possible third-party stakeholders.

In this study, we are interested in factors involved in all aspects of preparedness for mass gatherings from the rescue authorities’ point of view. According to previous research, mass gatherings cause an increased workload for emergency medical services (EMS), hospitals, and police forces [8,10,11,12]. According to Zeitz et al. [8], an increase in workload for rescue service operative resources is minor and their role in mass gatherings is characterized as being on standby in case of incident [8]. Different factors were shown to have an impact on the need of transport to hospital, the number of patients, and the injury patterns. These include weather [13,14], the level of the on-site medical care [15,16,17,18,19,20,21,22,23], audience profile [24], event type [25,26,27], duration [28], and geographical location [29]. Alcohol and drug use, attendance, and the age and mood of the crowd can also have an effect on the level and the need of medical response [28]. The history of disasters in mass gatherings represents various reasons for casualties, including overcrowding and crowd control issues, limited event access, issues in fire safety, limited medical preparedness, and limited emergency response [9]. In addition, weather and environmental hazards cause casualties in mass gatherings [30]. Previous literature from the viewpoint of preparedness includes research in predicting the resource use and the need for first aid services and transport to hospital [31,32,33]. Despite the fact that preparedness for mass gatherings and inspection of the emergency plans of events is the rescue authorities’ work on a daily basis as part of their legal duty of accident prevention, their own point of view regarding all aspects of preparedness for mass gatherings is missing from the literature.

In Finland, the largest mass gatherings are pre-planned festivals, concerts, and religious events. In addition to pre-planned events, some un-organized mass gatherings can occur, such as after major sports wins. Finland has large regional differences in geography, and the population is highly dispersed. In total, 4.3 million of the population total of 5.5 million live in two (South and West Finland) of the five former provinces, and all the big cities are located there [34]. The biggest mass gathering events also occur in that region, with the exception of singular annual festivals and itinerant religious events with large attendances that tend to take place in rural areas of the north. Additionally, most of the country has long distances as a fundamental geographical characteristic, which creates a further need to look at the phenomenon from the preparedness point of view.

In Finland, the rescue authorities play an essential role in preparedness for mass gatherings because of their legal duty of accident prevention. The Rescue section of the Ministry of the Interior of Finland coordinates the national Rescue service, whose duties and responsibilities are legislated in the Rescue Act 379/2011. Accident prevention is entrusted as one of the core tasks of the Rescue Service of Finland, and it is carried out in co-operation with other authorities, local communities, and residents. Actions for accident prevention additionally include surveillance, guidance, and advisory activities [35]. The Rescue service in Finland is divided into 22 regional rescue departments across the country [36]. At the departmental level, the specialists who represent a mix of job titles, including fire officers, inspectors, fire chiefs, risk management chiefs, and fire engineers, do their essential part in the processing work of emergency plans for mass gatherings. The authorities implement the planned mass gathering events’ emergency plans together with the police administration and the local hospital district. The pre-event planning work is intended to be done in close collaboration with the event organizer and stakeholders such as the on-site first=aid providers.

In summary, mass gatherings cause a need for pre-planning and preparedness for many actors via various mechanisms. The aim of this study was to investigate what factors, according to the rescue authorities, need to be considered when preparing for mass gatherings. This investigation of the details associated with the all aspects of preparedness for mass gatherings fills gaps in the research and enables further and deeper examination of the phenomenon.

## 2. Materials and Methods

The research material was collected by the first author, via semi-structured thematic interviews in 2019. The aim of the interviews was to identify all the factors that the rescue authorities need to consider when they are preparing for mass gatherings. The interview structure was based on previous studies on mass gatherings [8,11,13,14,15,23,25,37]. The original interview structure included background information and eight topics. The interview structure was revised during the data collection process when new points of view came up. This was done in order to gather all the possible information regarding the subject from informants. The results of this study divert a lot from the original interview structure. The informants (*n* = 15) were rescue service specialists across the country, whose work is associated with the topic; they represented a mix of job titles including fire officers, fire chiefs, rescue chiefs, risk management chiefs, and fire engineers. The recruitment of informants was carried out by sending an invitation to reach volunteer specialists from all (22) Rescue Departments, whose daily work involves preparedness for mass gatherings. The essential rescue departments from regions where the biggest mass gathering events occur were all represented.

The informants included both men (*n* = 12) and women (*n* = 3). Their age varied from 32 to 61 years and work experience from duties considering mass gatherings varied from 1.5 to 25 years. Educational backgrounds of the informants varied and included vocational, bachelor, and master level degrees. Participation in the study was voluntary, and the informants signed an informed consent form. The informants were aware that the material would be used for scientific purposes, and full anonymity was guaranteed throughout the whole research process and afterward.

The material consisted in total of 11 hours of interviews which were transcribed. The duration of the interviews varied from 24 minutes to 83 minutes. The average duration was 44 minutes. The transcribed data consisted of 195 pages in Finnish language. The material was anonymized prior to the analysis phase by ensuring the removal of names and any other personal information that could reveal the identity of the informants. Both the audio and written materials are stored in a safe location. The research permit for the study was obtained from all organizations that the informants represent. According to Finnish regulations, ethics committee approval was not needed. The ethical principles of University of Helsinki for research involving human subjects were followed [38]. 

**Figure 1:**
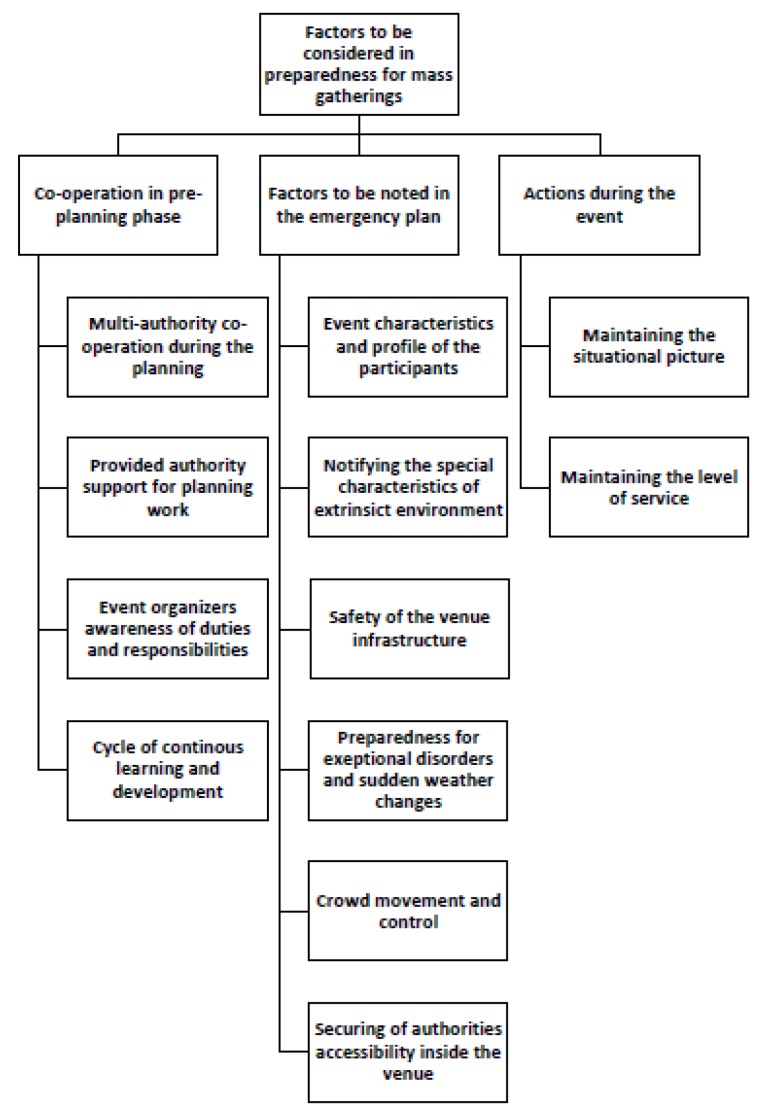
Main categories and generic categories.

**Figure 2:**
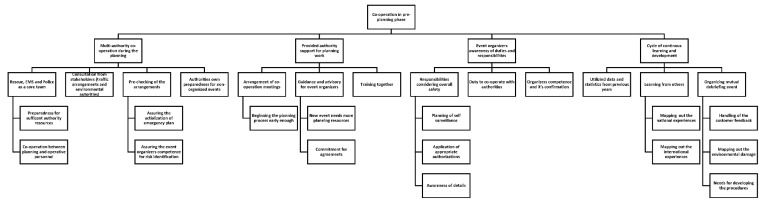
Co-operation in pre-planning phase and sub-categories.

**Figure 3:**
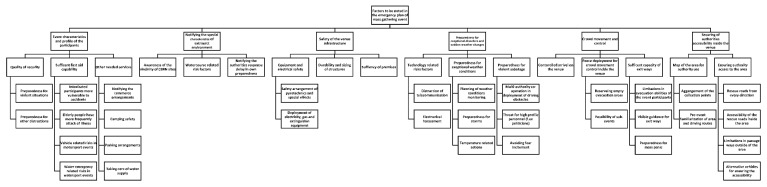
Factors to be noted in the emergency plan and sub-categories.

**Figure 4:**
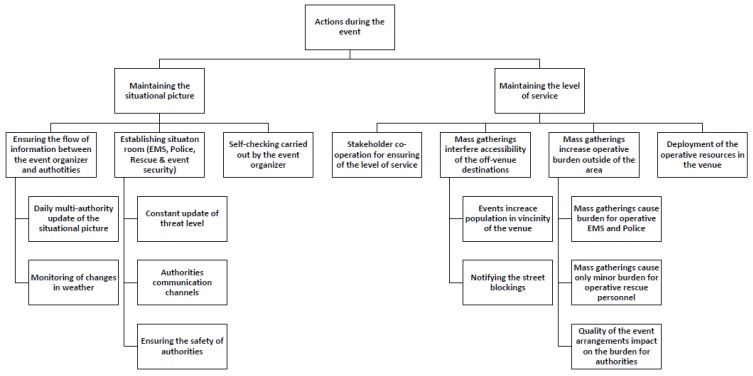
Actions during the event and sub-categories.

The analysis phase followed the inductive content analysis process described by Elo and Kyngäs (2008). The first author coded the material into meaningful headings and sentences, and the first and third authors then organized the coded and mixed material into similar groups, which were then headlined and categorized. The material formed three main categories with multiple generic and sub-categories (see Figure 1, Figure 2, Figure 3 and Figure 4). The forming of the categories was done in full agreement after collaborative discussion between the first and third authors.

## 3. Results

Three main categories emerged from the material: (1) co-operation in the pre-planning phase, (2) factors to be noted in the emergency plan, and (3) actions during the event. These main categories were divided into sub-categories (Figure 1), which were further divided into multiple generic categories (Figure 2, Figure 3 and Figure 4).

### 3.1. Co-Operation in the Pre-Planning Phase

The main findings that emerged from this category were multi-authority co-operation during the planning phase, provided authority support for planning work, event organizers’ awareness of duties and responsibilities, and the cycle of continuous learning and development (Figure 1).

Multi-authority co-operation in pre-planning means that the rescue authorities share information with their colleagues across the country. The threshold to consult each other is low. In addition, the preparedness for mass gatherings requires sufficient authority resources and collaboration between the administrative and operative sectors. Co-operation also includes consultation with the stakeholders regarding things such as traffic arrangements and environmental issues. Pre-checking of the arrangements guarantees the actualization of the emergency plan in the real world setting, and includes the confirmation that the event organizer has the competence for identification of potential risks. The authorities also need to take care of their own preparedness and ensure sufficient resources for the non-organized events.

*“One of the most essential things in the background of planning is the multi-authority co-operation.” (Informant 7)*

Provided authority support for planning work includes the arrangement of co-operation meetings where all the essential participants of the planning process gather together. In order to succeed in the pre-planning phase, the process, including the meetings, should commence early enough. The rescue authorities give guidance and advice for the event organizers according to their needs, as new events require more planning resources than long-running annual events with years of experience from the organizers. In addition, it is vital that the event organizer has full commitment for the arrangements made in the pre-planning phase. Rescue authorities and event organizers additionally collaborate in training together with different scenarios in order to make the actions more fluid in a possible real life situation during the event.

*“We are training for a disaster scenario in a rock festival environment, involving the event organizer, city officials, and key authorities. The actual situation leaders of organizations train together, and we are trying to find out how the co-operation is working, and how the communication actually works. We train with map scenarios and also have a mass casualty incident scenario in the actual venue.” (Informant 4)*

The event organizer’s awareness of their duties and responsibilities includes responsibilities concerning the overall safety, as well as the planning of self-surveillance during the event, and obtaining required permits and licenses. In addition, the event organizer should be aware of the details, including the estimated maximum number of participants in the area. The rescue authorities also need to make sure that the event organizer will be aware of the duty to co-operate with the authorities. The event organizer’s competence and the need for confirmation also surfaced from the material.

*“In the case of mass gatherings, we actually also make preparations, especially in securing of the EMS accessibility, although the main responsibility is primarily on the event organizer. Experience has taught us that we also need to be prepared.” (Informant 1)*

The cycle of continuous learning and development includes utilization of data and statistics from the previous years in order to forecast the need of operative resources. In addition, learning from others and mapping out the national and international experiences strengthens the level of preparedness as the lessons learned are implemented in practice. Organizing a joint debriefing after the event includes handling of the customer feedback and mapping out the environmental damage. It also enables mapping of the need for developing the resources in the future.

*“All information from the past years, including the international experiences, are taken into account and considered whether it could happen here. The lessons learned are implemented into our guidelines and requirements and it is also ensured that the event organizer is aware of them.” (Informant 7)*

### 3.2. Factors to Be Noted in the Emergency Plan

This main category is divided into sub-categories of event characteristics and profile of participants, notifying the special characteristics of the wider environment, safety of infrastructure, preparedness for exceptional disruptions and sudden weather changes, crowd movement and control, and securing of authorities’ access to the venue (Figure 3).

Event characteristics and profile of the participants include the quality of the security, which contains preparedness for violent situations and distractions such as rioting caused by some football fans. In addition, it includes sufficient first-aid capability that is divided into perception of intoxicated participants being more vulnerable to accidents and into elderly people experiencing more sudden onset of ill health. Moreover, motorsport events have vehicle-related risks and watersport events have water emergency-related risks that in turn have an effect on the need for first-aid capability. The category of other needed services includes notifying the retailers in the event area, as well as details considering camping safety. Furthermore, parking arrangements and taking care of the water supply need to be considered in the emergency plan.

*“The event characteristics have a direct impact on the level of preparedness required from the event organizer. The number of people is actually not the essential thing, but it is the profile of participants and their conditions.” (Informant 8)*

Taking into account the special characteristics of the wider environment includes the awareness of CBRN (chemical biological radioactive nuclear) sites such as industrial facilities in the vicinity of the event site. The watercourse-related risk factors might also require special preparedness from the event organizer, such as surface rescue capability. Additionally, the general awareness of the environmental characteristics’ impact on the authorities’ accessibility to the site needs to be considered in the organizers’ own preparedness actions.

*“In the events that occur in the vicinity of the watercourse that may involve risk of surface rescue or drowning, we might require surface rescue preparedness from the event organizers. Also, if the event is happening in cross-country or austere environments, we have required off-road rescue capability from the organizer, in order to avoid burden on our operative resource.” (Informant 8)*

The safety of the venue infrastructure includes taking care of equipment and electrical safety, including safety arrangements considering pyrotechnics and special effects and deployment of electricity, gas, and extinguishing equipment. The durability and sizing of the structures and sufficiency of premises needs to also be taken into consideration in the emergency plan.

Preparedness for exceptional disruptions and sudden weather changes includes technology related risk factors that contain distractions in telecommunications and electronic interference of the venue.

*“It is important to bring the operative resource to the scene in advance. People are inclined to use the emergency number, but in mass gatherings it won’t help because connections are down; even though the telecom operators bring extra access points and other equipment, they will eventually fail.” (Informant 2)*

Preparedness for exceptional weather conditions involves planning for the monitoring of the weather conditions and actions taken in case of storms and temperature-related issues. The category additionally includes preparedness for violent sabotage, such as terrorist attacks, which requires multi-authority co-operation, such as the deployment of driving obstacles to prevent the use of vehicles as weapons. Threats to high-profile personnel, for example, politicians, also needs to be taken into consideration in the planning. However, the rescue authorities pointed out that avoiding the fear incitement in preparedness for violent sabotage is important.

“In our co-operation with police, we have recently discussed, more than before, about the threat of an intentional attack. The threat of terrorism is constantly floating above us.” (Informant 3)

Crowd movement and control requires controlled arrival at the site and planning of the fence deployment in order to control the crowd movement inside the venue.

*“It is essential to control the crowd and its movement. In general, how the crowd is smoothly guided into the venue and out of there. It is a big issue, one where the problems usually encapsulate. If a big crowd suddenly packs into one place, and there is no preparedness to handle the crowd swiftly, the bottleneck is there.” (Informant 1)*

Possibility of sub-events, such as concerts inside the venue, need consideration, and empty evacuation areas should be reserved inside the venue in order to avoid evacuation in already crowded areas. The sufficient capacity of exit ways needs securing, and the limitations in evacuation abilities of the participants need to be noted. The exit ways should be visibly guided and the organizer should be prepared for mass panic.

*“If you already have the walkways and rescue roads full of people, you can’t push another three, four or five thousand extra people into those areas in a couple of minutes. Evacuation needs to be done to empty clear space, it is important.” (Informant 7)*

Securing of the authorities’ accessibility inside the venue requires a map of the area for the authorities, including arrangements of the collection points, and pre-event familiarization of the area and driving routes. In addition, the authorities’ access to the area needs to be confirmed by rescue roads from every direction to the venue and accessibility of the rescue roads inside the area. Limitations in passageways outside of the area need notification, and alternative vehicles, such as bicycles, can be used for ensuring accessibility.

*“An up-to-date location map provided by the event organizer is one of the most important tools for us, EMS, and police. We can find the exact position and correct gate to the area according to the pre-numbered locations in the map.” (Informant 7)*

### 3.3. Actions during the Event

The actions during the event sub-category was split into two generic categories of maintaining the situational picture and maintaining the level of service (Figure 4). In order to maintain the situational picture, the flow of information between the event organizers and the authorities needs to be ensured by daily multi-authority updates and constant monitoring of changes in the weather. Establishing a situation room for the key players including EMS, police, rescue service, and event security participants strengthens the situational picture by constant update of threat level, authorities’ communication channels, and ensuring the safety of the authorities. Self-checking carried out by the event organizer is also a vital factor in maintaining the situational picture.

*“Considering the rock festival in our area, we make an agreement about our situational leadership effort on the scene. We have our own situational leader, EMS situational leader, and operative resources on the scene during the whole event, and they collaborate with the event security organization.” (Informant 4)*

Maintaining the level of service requires co-operation between the stakeholders involved. Mass gatherings may interfere with the accessibility of the off-venue locations as the events increase population in the vicinity of the venue and streets might be blocked because of the event.

*“Most of the emergency calls during the event occur from outside of the venue, because the actions inside the event are so well-shaped along the years, and we can keep it in control. When people travel from the venue to the city center after the concerts, it creates a big peak in emergency calls.” (Informant 4)*

Additionally, mass gatherings increase operative workload outside of the event area.

*“Mass gatherings are a part of the modern society and belong inside the entirety of the level of service.” (Informant 2)*

*“We must think about enhancing our own emergency readiness during events. We cannot respond to challenges that these tens of thousands of additional people cause to our normal resources. We need to prepare for it.” (Informant 4)*

However, workload for the operative rescue personnel (rescue service) is only minor, as the major load builds up for EMS and police forces.

*“Most of the workload for authorities caused by mass gatherings builds up to EMS.” (Informant 1)*

The quality of the event arrangements such as the level and amount of first aid and security personnel influences the operative workload for the authorities. The deployment of the operative resources in the venue has an effect on the level of service in the area and improves the preparedness for major incidents.

*“Being already on the scene does not guarantee that nothing will happen, but if shit hits the fan, we can act immediately and don’t have to drive against the streaming crowd, which would cause more delay. In mass gatherings, we bring our own situation center and the appropriate number of units into the venue beforehand, in order to act immediately and to avoid the situation worsening because of delay.” (Informant 2)*

## 4. Discussion

The aim of this study was to identify factors that need to be considered in preparedness for mass gatherings from the rescue authorities’ point of view. Our findings showed that the rescue authorities pinpointed a broad range of factors involved with the preparedness for mass gatherings, covering the whole event from planning to execution. The factors involved were divided into three main categories: co-operation in the pre-planning phase, factors to be noted in the emergency plan, and actions during the event.

### 4.1. Co-Operation in the Pre-Planning Phase

The results showed that co-operation in the pre-planning phase is vital. Multi-authority co-operation during the planning is important in order to ensure the seamless collaboration between the authorities and stakeholders during the event. According to previous research, planning of mass gathering is significant for healthcare [39] and also requires co-operation with police forces and other emergency workers [37]. According to our results, multi-authority collaboration in the pre-event planning phase manifests, for example, as consultation of an EMS specialist regarding the planned attendee numbers, and the competence of the on-site medical care provided in the emergency plan. Previous studies showed that having medical professionals available among the first-aid providers and the use of treat-and-release directives have an impact on the need for transport to hospital, and in turn affect the workload for the EMS system and healthcare facilities [15,16,18,21,23]. According to an Australian study by Zeitz et al. [8], the increase in workload for the rescue service in mass gatherings with a total of 5.7 million attendees in a two-year period, was only minor. The workload for rescue service consisted of assistance in traffic control and on-call support. Police workload correlated with the EMS workload. The main determinant for the police force’s workload was weather, while EMS workload had a wider range of determinants. 

The need for authorities to prepare for mass gatherings with sufficient resources and the need for preparedness for non-organized events, such as nationwide celebrations after major sports wins or end of school celebrations in May also stood out from the results. Previous research shows that mass gatherings may especially strain resources of the EMS, healthcare facilities, and police forces, and they require pre-planning [8,12,37,40]. The results of this study further showed that pre-checking of the arrangements ensures the actualization of the emergency plan and event organizer’s competence for risk identification. It is also vital that the authorities provide support for the event organizer in the pre-planning phase. In addition, the event organizer should be aware of their duties and responsibilities. Issues that can be checked in the pre-planning phase, such as fire safety protocols, the availability of fire exits, pre-event tabletop exercises, communication with EMS, the availability of access routes, and the dereliction of organizer duties were identified as learning points from the mass gathering disasters in the past [9]. The importance of the cycle of continuous learning and development was evident in the results. Previous literature showed that retrospective analysis and examination of the collected data from past years’ mass gatherings reveal correlation of patient presentation and transport to hospital rate with different variables, such as size of crowd, day of week, temperature, and humidity; thus, they can be used as tools for forecasting the need for medical assistance [31,32,41,42].

### 4.2. Factors to Be Noted in the Emergency Plan

Previous studies indicated that understanding audience motivations makes it possible to gain an accurate picture about how the motivations might impact the crowd behavior [24]. Understanding crowd behavior in turn allows adequate crowd management assessment in the pre-event phase [43]. In addition, event characteristics, such as the event type, alcohol and drug use, and weather affect the need of medical attention and injury profiles [8,21,25,26,27,28,42,44]. Our results showed that awareness of event characteristics and the profile of the participants are vital factors to be noted in the emergency plan of a mass gathering event. Intoxicated participants are more vulnerable to accidents [28]. Intoxicated patrons might also cause elevated workload for police forces [8]. Sufficient first-aid capability and the quality of security emerged from the results along with other needed services, such as taking care of water supply. Previous studies indicated that on-site medical staff should include health professionals along with first-aid providers [15,18,19,23,40]. According to Locoh-Donou et al. [26], absence of free drinking water is strongly associated with patient presentations. According to Bledsoe et al. [29], special attention needs to be paid to preparation and medical care, when a large event is held outdoors in an austere and remote environment [29]. Our findings support this, as the authorities pointed out that the special characteristics of the extrinsic environment should be included in the event emergency plan. This includes notifying the authorities of any delays in response when organizing events in austere environments, thus also requiring actions on the level of event organizer preparedness.

According to our findings, safety of the venue infrastructure was seen as a factor that should be included in the emergency plan. Notifying the equipment and electrical safety, durability and sizing of structures, and sufficiency on premises support the lessons learned from the past. Structural deficiencies caused disasters in the history of mass gatherings, [9,45]. In addition, insufficiency of premises caused many disasters through overcrowding and led to multiple deaths and injuries [9,46].

Based on our results, preparedness for exceptional disorders, such as violent sabotage and electronic harassment and distractions of telecommunication, should be taken into account in the emergency plan. Furthermore, preparedness for exceptional weather conditions should be included in planning. An elevated threat for high-profile personnel also emerged from the data. The Finnish National Risk Assessment 2018 indicates that, in addition to traditional threats, mass gatherings cause a need for preparedness in terms of the counter-terrorism aspect, as large crowds and public places were identified as potential targets for an act of terrorism [7]. According to previous studies, potential violent sabotage may occur unpredictably, lacking systematic characteristics, and the attackers may lack clear objective, which means that their action might be less rational compared to traditional terrorists, in a case such as the Boston marathon bombings in April 2013 [47]. The risk for terror strike is elevated if the event involves the attendance of politicians or other high-profile targets. These events may create an increased risk for use of explosive devices or assassination attempts [48]. In addition, our findings indicate that exceptional weather conditions create a need for preparedness, planning of weather condition monitoring, and temperature-related actions. This is supported in previous literature as, according to a review by Somaroo and Murray [30], extreme weather conditions created many learning points from fatal structural collapses caused by storm to high temperatures causing heat-related illness [30].

Issues in crowd control caused numerous disasters in the history of mass gatherings. This point also stood out from our findings as a factor to be included in the emergency plan. According to Soomaroo and Murray [9], in the Love Parade disaster at Duisburg, Germany in 2010, one of the potential causes was insufficient exit ways and issues in crowd control. This was also a case in the 2001 night club fire in Volendam, Netherlands. In the 2000 Roskilde festival disaster, nine people died in crowd surge and crushing in a mosh pit during a rock concert. In 1971, 66 people died and 140 were injured when exiting and entering fans were crushed in a football stadium in Glasgow, Scotland [9]. In our results, the details considering crowd control included controlled movement in and out of the venue, appropriate crowd control inside the venue, and a sufficient capacity of the exit ways. Securing the authorities’ accessibility inside the venue was similarly pointed out. Accordingly, the absence of EMS access routes was identified as learning points in mass gathering disasters of the past [9].

### 4.3. Actions during the Event

In our results, actions during the event require maintaining the situational picture and the level of service. Ensuring the flow of information between the event organizer and the authorities is important, and poor communication with EMS was indeed a factor in many mass gathering disasters [9]. Information flow considering monitoring of weather changes is also important in order to ensure the capability for mass evacuation. Previous literature identified cases where mass evacuation due to extreme weather conditions was carried out [30]. Our findings suggest that establishing a situation room enables a constant update of the threat level, ensures the authorities’ own safety, and shifts communication between the authorities and event security officials. In addition, the absence of an operation command center was pinpointed as learning point in the Ellis Park 2001 disaster [9]. Our results showed that self-checking during the event carried out by the event organizer creates a continuum with and builds up from the pre-checking carried out by the authorities. Maintaining the level of service during the event involves accessibility to off-venue destinations, awareness of increased workload caused by mass gatherings outside of the area, and the deployment of operative resources in the venue. According to previous studies, mass gatherings mainly increase workload for the EMS, healthcare facilities, and police forces [5,6,8,10,11,12,14,15,16,20,21,25,26,27,28,29,31,48,49,50,51]. This supports the findings of the present study, as the informants pointed out that operative workload during the event builds for EMS and police, whereas the increase in workload for rescue service operative personnel is only minor.

### 4.4. Strengths and Limitations

The informants represented a mix of job titles with the unifying factor of working with the topic of this study—preparedness for mass gatherings. The interviewed specialists had years of experience working in the rescue service and, thus, obtained advanced-level training, such as an engineering degree or rescue officer studies. The informants represented different geographical parts of the country from the capital metropolitan area to small provinces of North Finland. Informants from the areas where the biggest mass gatherings occur were represented. Specialists who work with the subject in Finland are few, and the informants of this study represented a comprehensive sample of this group.

The categorization of the coded interview material was done in collaboration by two researchers in order to increase trustworthiness of this study. The original raw data were in Finnish, and categories and direct quotations included in the article were translated into English after forming the categories. As the data involved spoken language in written form and the informants occasionally used colorful language, some of the quotes were challenging to translate. Although the core message of the translations remained unchanged, there might be some bias in the translated quotations compared to the actual words of the raw data.

## 5. Conclusions

The rescue authorities recognized various factors considering preparedness for mass gatherings. These factors can be categorized into co-operation in the pre-planning phase, factors to be noted in the emergency plan, and actions during the event. Mass gatherings are part of society and belong inside the level of service. In order to ensure sufficient operative resources, such as the ability to deploy of operative resources beforehand in the venue in order to improve disaster preparedness, sufficient financial resources are required. The operative workload increase caused by mass gatherings concentrates especially for the EMS and the police, whereas, for rescue service operative troops, the increase in workload is only minor. It should also be noted that the workload for operative resources occurs outside of the actual venue and peaks at certain times of day. This phenomenon should be studied more closely. In addition, further research is required through geographical information systems (GIS) and dispatch profiles. By obtaining precise information regarding the amount, time, and place of the operative workload, more accurate pre-planning is enabled, as well as more effective use and deployment of the limited operative resources.
